# Public health system challenges in the Free State, South Africa: a situation appraisal to inform health system strengthening

**DOI:** 10.1186/s12913-019-4862-y

**Published:** 2020-01-23

**Authors:** B. Malakoane, J. C. Heunis, P. Chikobvu, N. G. Kigozi, W. H. Kruger

**Affiliations:** 1 Department of Community Health, University of the Free State, P.O. Box 339, Bloemfontein, 9300 South Africa; 2 Centre for Health Systems Research & Development, University of the Free State, P.O. Box 339, Bloemfontein, 9300 South Africa; 3 Free State Department of Health, P.O. Box 277, Bloemfontein, 9300 South Africa

**Keywords:** Free State, Public health system, Public health system challenges, WHO building blocks, Causal loop diagram, Fragmentation, Integration

## Abstract

**Background:**

Since the advent of democracy, the South African government has been putting charters, policies, strategies and plans in place in an effort to strengthen public health system performance and enhance service delivery. However, public health programme performance and outcomes remained poor while the burden of disease increased. This was also the case in the Free State Province, where major public health system challenges occurred around 2012. Assessment was necessary in order to inform health system strengthening.

**Methods:**

The study entailed a multi-method situation appraisal utilising information collated in 44 reports generated in 2013 through presentations by unit managers, subdistrict assessments by district clinical specialist teams, and group discussions with district managers, clinic supervisors, primary health care managers and chief executive and clinical officers of hospitals. These data were validated through community and provincial health indabas including non-governmental organisations, councils and academics, as well as unannounced facility visits involving discussions with a wide range of functionaries and patients. The reports were reviewed using the World Health Organization health system building blocks as a priori themes with subsequent identification of emerging subthemes. Data from the different methods employed were triangulated in a causal loop diagram showing the complex interactions between the components of an (in) effective health system.

**Results:**

The major subthemes or challenges that emerged under each a priori theme included: firstly, under the ‘*service delivery*’ a priori theme, ‘*fragmentation of health services*’ (42 reports); secondly, under the ‘*health workforce*’ a priori theme, ‘*staff shortages*’ (39 reports); thirdly, under the ‘*health financing*’ a priori theme, ‘*financial/cash-flow problems*’ (39 reports); fourthly, under the ‘*leadership and governance*’ a priori theme, ‘*risk to patient care*’ (38 reports); fifthly, under the ‘*medical products/technologies*’ a priori theme, ‘*dysfunctional communication technology*’ (27 reports); and, sixthly, under the ‘*information*’ a priori theme, ‘*poor information management*’ (26 reports).

**Conclusion:**

The major overall public health system challenges reported by stakeholders involved fragmentation of services, staff shortages and financial/cash-flow problems. In order to effect health systems strengthening there was particularly a need to improve integration and address human and financial deficiencies in this setting.

## Background

Chronic underfunding of public health has negatively influenced the ability of existing health systems in African countries to respond to healthcare needs [1], p. 249. In South Africa, despite increases in public healthcare utilisation due to the high burden of disease and increased patient load over the period 1997–2010, the public health sector fell from second to fourth in the list of spending priorities [2]. Consequently, major health system challenges occurred in this sector including negative staff attitudes, long waiting times, unclean facilities, medicine stock-outs, insufficient infection control and compromised safety and security of both staff and patients [3]. Poorly performing health systems may fail to contain epidemics, as was observed in three West African countries, Guinea, Sierra Leone and Liberia where there was an Ebola outbreak in 2014 [4, 5]. The comprehensive economic and social cost of the Ebola epidemic has been estimated at $53.19 billion (2014 USD) [6].

As defined by the World Health Organization (WHO), health systems comprise of all the organisations, institutions and resources that are devoted to producing actions whose primary purpose is to improve health [7]. The overall vision of a public health system is “*to promote greater health and well-being in a sustainable way, while strengthening integrated public health services and reducing inequalities*” [8]. Public health systems are thus “*more than the pyramid of publicly owned facilities that deliver personal health services*” [9], p. 2 and can more comprehensively be defined as “*all public, private, and voluntary entities that contribute to the delivery of essential public health services within a jurisdiction*” [10].

The WHO further defines overall health system outcomes or goals as “*improving health and health equity, in ways that are responsive, financially fair, and make the best, or most efficient, use of available resources*” [9], p. 2. Health system performance is variably measured across dimensions such as quality, cost, access, equity, and patient experience and safety [11]. Health system strengthening refers to activities to improve a country’s ability to successfully perform the essential functions described by the WHO’s six health system components or building blocks: service delivery; healthcare workforce; information; medical products, vaccines and technologies; financing; and leadership and governance [9]. Given the importance of the public healthcare sector for a country’s epidemic preparedness and efforts to achieve universal health care (UHC), the public health system needs to perform well in respect of each of the six health system building blocks.

Over the last three decades, health systems around the world have faced difficulties such as urbanisation and changes in behaviours that contributed to the pervasiveness of chronic diseases, unrelenting high rates of infectious diseases, and rising rates of traumatic injuries, violence and road traffic accidents [12]. Improving the public health system’s efficiency as a means to reach UHC [13] is urgently needed in South Africa. The country is facing a quadruple burden of disease including a dual HIV-TB epidemic (about 17% of global burden), high maternal and child mortality (about 1% of global burden), high levels of violence and injuries (about 1.3% of global burden), and increasing non-communicable diseases (about 1% of global burden) [14]. In the Free State Province, mental health is considered a fifth additional burden [15]. The country’s disease profile and burden is closely related to enduring developmental inequalities and inequities resulting from its apartheid history. As reported by the World Bank, the Gini index for income inequality – an indicator for achieving Sustainable Development Goal 10, to reduce inequality within and among countries – for South Africa in 2011 was 0.634 [16]. In the same year, 16.6% of the country’s population lived below a poverty headcount threshold of $1.90 a day [17].

While South Africa spends more on health than any other African country, health outcomes are not commensurate with spending [18]. A study on measuring overall health system performance for 191 WHO member states in 2000, ranked France as the best, while South Africa was ranked 175th [19]. This low-ranking position for the country underscored the need for public health system strengthening to enhance the overall performance of the health system and contribute to improve the welfare and life expectancy of the population.

Disease-specific programmes in South Africa are structured within public healthcare system organograms as directorates or programmes that are semi-independently managed through the district health system (DHS). This has created ‘*pseudo-specialisation*’ [20] where managers only focus on their respective programmes and specialities without considering nor communicating any synergies between their own programmes and others, thereby resulting in narrowing interactions across the programmes. Decisions are typically made centrally and then have to be implemented lower down at district level with central monitoring and control being exercised from head office. Fund allocations from the national budget take the form of either conditional (programme-specific) fund allocations or appropriated (equitable share) fund allocations. The limited interactive arrangement of programmes inhibits consultation and sharing of knowledge among the officials (healthcare managers and healthcare workers [HCWs]) and other stakeholders. This situation was also experienced in the Free State at the time of the situation appraisal.

One of nine provinces in South Africa, the Free State accommodates 5.1% of the public health sector dependant population of whom more than 80% are African [21] and historically and socioeconomically disadvantaged due to apartheid spatial and homeland planning [22], inequality in public funding allocation [23], social exclusion and segregated access to public sector amenities [22, 24]. At ZAR 91 994 ($6 822), the average annual household income in the Free State in 2010/2011 was substantially lower than that of the country as a whole at ZAR119 542 ($8 865) [25]. In 2012, the Province had the third lowest life expectancy for males (53.0 years compared to 61.5 years in the Western Cape Province) and the fourth lowest life expectancy for females (59.9 years compared to 67.5 years in the Western Cape Province) [26], p. 217.

The South African health system is made up of the public and private health sectors, with the former being operated by the various provincial government health departments. The public health sector is divided into primary, secondary and tertiary health services provided through various health facilities located within and managed by the different provincial departments, under monitoring by the National Department of Health [27]. Since its advent to power in 1994, the new South African government has put a succession of charters, policies, strategies and plans in place in an effort to strengthen public health system performance and to enhance health service delivery [28]. In addition, the national and provincial governments have increasingly adopted the WHO building blocks approach in assessing public health system performance [29]. In spite of all the plans and strategies and regulatory and policy interventions, and despite a clear agenda for quality health care and significant annual expenditure, public health system shortcomings continue to endanger the health and lives of South Africans and have resulted in a loss of confidence among users [30].

A review of mortality trends and differentials across South Africa covering the years, 1997 to 2012 highlighted serious public health system challenges in the Free State as reflected by the higher mortality rates due TB, HIV/AIDS and lower respiratory tract infections compared to the national rates [31]. More specifically, the proportion of deaths attributable to HIV in the Free State was 36.1% as compared to 35.7% in South Africa at large, the proportion of deaths attributable to TB was 4.9% compared to 4.6% nationally, and the proportion of deaths attributable to lower respiratory tract infections was 6.5% compared to 4.6% nationally. A review of data from the District Health Information System (DHIS) (2015), indicates that immunisation coverage amongst children in the Free State declined from 97% in 2011 to 87% in 2014 [32]. The caesarean section rate increased from 22% in 2011 to 25% in 2014, thereby indicating the possibility of system deficiencies from antenatal care up to delivery. The tertiary hospital bed utilisation rate increased from 73% in 2011 to 93% in 2014. This could have indicated challenges in the ability of the lower levels of care to intervene timeously and appropriately in order to prevent health complications. While the TB cure rate improved from 73% in 2011 to 76% in 2013, it again declined to 75% in 2014.

These patterns and trends suggest that there was a general decline and widespread weaknesses in the Free State’s public health system. This situation appraisal sought to investigate healthcare managers, frontline service providers and community stakeholders’ perspectives of the health system challenges related to poor public healthcare service delivery in order to inform health system strengthening in this context.

## Methods

### Setting and design

The Free State has an estimated population of 2.87 million of which about 80% are dependent on public health services. The study entailed a multi-method appraisal [33] of information collected in the form of unit managers’ presentations, stakeholder group discussions and validated in community and provincial level ‘*indabas*’ (meetings convened to address a particular matter, in this instance the provincial or local state of public health services), during a process of provincial executive leadership transition in 2013.

### Population and sampling

The study population broadly consisted of the public health service providers and community members in the Free State. The healthcare providers that participated in the data gathering were purposefully selected based on their role in the public health provisioning system, including unit managers, district clinical specialist teams (DCSTs), subdistrict managers, clinic supervisors, and primary health care (PHC) and hospital managers (details in Step 2 below). The selection of community members who participated in the validation of the data was also purposive; i.e. persons who attended the community and provincial indabas and those present (including patients) at unannounced visits to public healthcare institutions across the Province (details in Step 3 below).

### Data gathering and problem identification

**Fig. 1:**
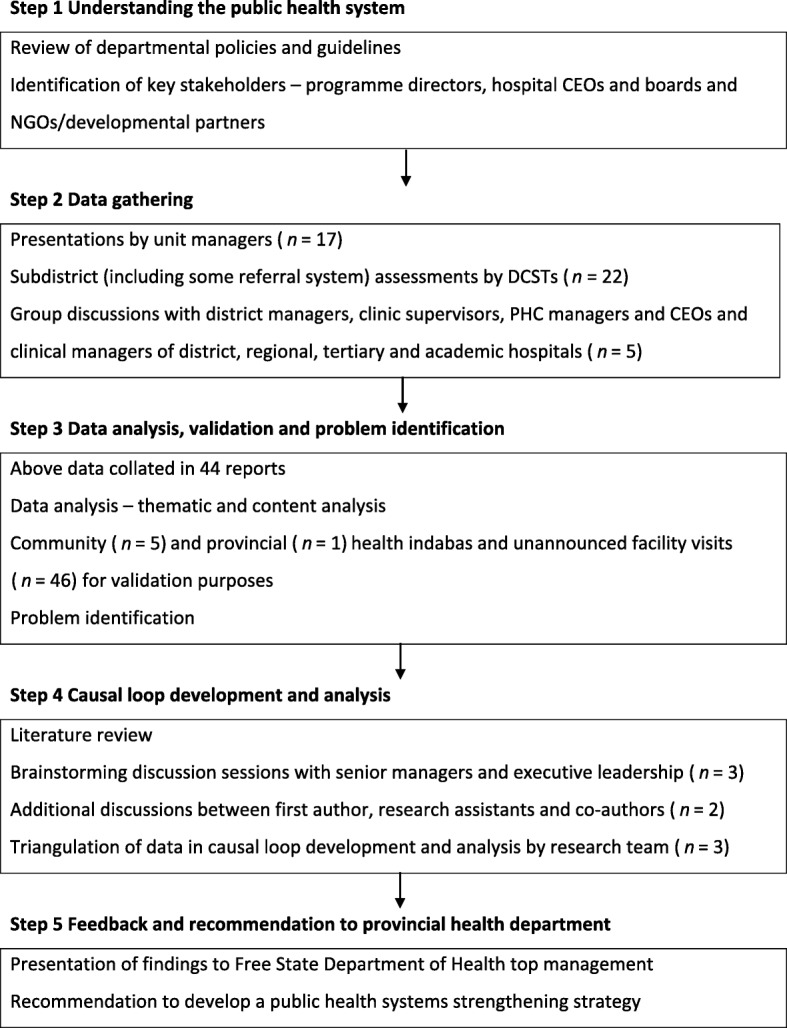
Research design and problem identification procedure

Figure 1 outlines the data gathering and problem identification procedure.

#### Step 1 Understanding the health system

At the outset, a review of departmental policies and guidelines was conducted. Thereafter, key stakeholders, including programme directors, hospital chief executive officers (CEOs), hospital board members and non-governmental organisation (NGO)/development partner representatives were identified.

#### Step 2 Data gathering

Management consultative workshops and meetings were held during which 17 presentations about the functions and status of every departmental component were delivered by line managers, programme managers, district managers, and CEOs of hospitals. A structured presentation format, informed by guiding documents including the component organograms and relevant strategies and policies, was followed in each presentation.

In order to gain deeper understanding of the situation and material conditions that existed within public healthcare facilities, the district clinical specialist teams (DCSTs) were tasked to visit the subdistrict management teams (local area and clinic operational managers) to investigate the key issues related to service implementation. The full complement of available DCSTs and their nurse equivalents was used to this end. Although the DCSTs were not complete in their composition, it is thought that they were sufficiently representative to fulfil this task. The DCSTs were required to make recommendations based on their observations.

To fully understand the functioning of the referral system throughout the DHS, 22 separate group discussions were also conducted by the DCSTs with subdistrict managers, clinic supervisors, PHC managers and the CEOs and clinical managers of district, regional, tertiary and academic hospitals covering all districts. The responsibilities of district hospitals and PHC clinics are interrelated since the hospitals receive upward referrals from clinics and downward refer back to them. The same format of data gathering was followed as used by the unit managers in their presentations. The main purpose of the group discussions was to understand the interrelationships and cooperation in the handling of the upward and downward referral between different levels of care across the DHS. Notes were then taken by the DCSTs during each group discussion.

The purpose of the above-mentioned activities was to closely identify challenges negatively affecting public health service delivery in the Free State. A total of 44 reports were generated based on these activities and were reviewed for the purposes of this paper.

#### Step 3 Data analysis, validation and problem identification

The review of the reports was conducted in line with the six WHO building blocks as a priori themes. Thematic analysis was used to synthesise the emerging subthemes or challenges. Textual information was first sorted according to the a priori themes. Thereafter, emerging subthemes were identified and manually coded. Firstly, two coders independently reviewed each report and identified and grouped similar responses into subcategories. Secondly, the coders compared notes and resolved any differences that showed up on their initial categorisation to ensure that the categories were independent, mutually exclusive and exhaustive. Thirdly, the first author used the consolidated list of generic categories to independently code the data. The first author and support staff independently verified the alignment of the emerging subthemes. The coded information was transferred to STATA 12 [34] to generate frequency counts of the cited public health systems challenges.

These data were validated through five community level and one provincial level health indaba including community members, NGOs, AIDS councils and academics, as well as 46 unannounced facility visits. During the latter visits, the first author and his support team independently had discussions with hospital CEOs and managers, clinic managers, HCWs, pharmacists and pharmacist’s assistants, filing clerks, data capturers, maintenance and security staff, volunteers, laundry workers, kitchen staff, emergency medical services (EMS) staff, and, importantly, patients themselves. These discussions served the purpose to validate and further understand the subthemes or challenges raised in the 44 reports analysed prior to development of the causal loop diagram.

#### Step 4 Causal loop diagram development and analysis

Causal loop (influence) diagrams (CLDs) are circular chain diagrams of cause and effect that are used to represent complex and non-linear interactions among variables which are often difficult to describe. A relationship between two variables is represented by an arrow showing the direction of influence. A positive sign on a link implies that a change in one variable results in a change in the same direction, whereas a negative sign denotes a change in the opposite direction. A feedback loop occurs when arrows connect a variable to itself through a series of other variables. A feedback loop may be reinforcing (R) or balancing (B). A reinforcing loop is defined as a positive feedback system that represents a growing or declining action, while a balancing loop is a negative feedback system that is self-regulating [35]. The CLD is thus a qualitative representation of the underlying mental models that emerged during the review of reports. Presenting the health system dynamic using CLDs has been used in other studies [36, 37].

In order to understand the interrelationships and dynamics of the public health system, brainstorming discussions between senior managers and the executive leadership of the provincial health department took place to develop the CLD. This was followed by additional discussions between the first author, research assistants and co-authors. The CLD was developed using Vensim PLE software for systems dynamics modelling [38].

Construction of the causal loop was informed by literature study, as well as the research team’s engagements with various experts from the provincial health department including information from the brainstorming discussions. So-doing, the interrelationships between the identified emerging subthemes were used to illustrate the complex interactions among the components of an (in) effective health system. In the current study, the CLD was also used to triangulate and better understand the rich data (system-wide challenges) arising from the 44 original reports, the six indabas and the 46 unannounced facility visits.

#### Step 5 Feedback and recommendation to provincial health department

The results were presented to the provincial health department’s top management and it was recommended that a health system strengthening strategy should be developed. The development, implementation and evaluation of this strategy will be presented elsewhere.

### Ethics

Although most data were gathered through routine departmental management processes, subjects were informed that the data was also going to be used for research purposes. No names or other identifying information are disclosed in the reporting of the findings. The Health Sciences Research Ethics Committee (Institutional Review Board number: IRB00006240) at the University of the Free State approved the study.

## Results

The emerging subthemes or challenges under each of the a priori themes are indicated in Table 1.

**Table 1:** Emerging subthemes per health system building block

A priori theme	Emerging subtheme	Number of reports	Percent (*n* = 44)
Service delivery	Fragmentation of services	42	95.5
	Poor quality of service	29	65.9
	Infrastructure challenges	29	65.9
	No institutional road signs	22	50.0
	Poor security in the institutions	5	11.4
	Poor referral system	2	4.5
	Dysfunctional EMS	2	4.5
Healthcare workforce	Shortage of staff	39	88.6
	Poor HRH management	38	86.4
	RWOPS challenges	18	40.9
	Shortage of accommodation for interns	20	45.5
Information	Poor information management	26	59.1
Medical products, vaccines and technologies	Dysfunctional ICT system	27	61.4
	Shortage of resources/equipment	26	59.1
	Pharmaceutical system challenges	23	52.3
	Supply chain management challenges	8	18.2
Financing	Financial/cash flow challenges	39	88.6
	OSD challenges	3	6.8
Leadership and governance	Risk to patient care	38	86.4
	Poor leadership	29	65.9
	Poor priority setting	27	61.4
	Lack of governance structures	22	50.0
	Lack of policy implementation	8	18.2
	Non-appointment of hospital board members and clinic committee members	1	2.3

### Service delivery

Seven emerging subthemes were identified under this a priori theme including: firstly, ‘*fragmentation of health services*’ (42 reports); secondly, ‘*poor quality of service*’ (29 reports); thirdly, ‘*infrastructure challenges*’ (29 reports); fourthly, ‘*lack of institutional road signs*’ (22 reports); fifthly, ‘*poor security in institutions*’ (9 reports); sixthly, ‘*poor referral systems*’ (2 reports); and, finally, ‘*dysfunctional EMS*’ (2 reports). Challenges relating to service delivery were echoed in several group discussions. For example, a district manager mentioned that “[t]*here has been various incidents of theft affecting our clinics. At one clinic, the burglar bars and window were broken and the thieves gained entry via the sluice room and got away with two computers. This concern is due to shortages of security guards at some facilities, with some not having any security at all. This puts both the safety of both staff and the patients at risk.*” At one indaba, some community members highlighted that while post-apartheid South Africa has displayed political commitment to healthcare reform, the country does not yet have adequate infrastructure within its different types of facilities to cope with the growing disease burden and service demands. Community members further raised concerns about poor attitudes of staff members towards patients, for instance: “[a]*t times* the *attitude of people answering the EMS call centre is not good. They are rude and do not seek to humanely understand the reason for your call.*”

### Healthcare workforce

Four emerging subthemes were identified under this a priori theme including: firstly, ‘*healthcare staff shortages*’ (39 reports); secondly, ‘*poor HRH* [human resources for health] *management*’ (38 reports); thirdly, ‘*high vacancy rates*’ (33 reports); and, fourthly, ‘*RWOPS* [Remunerative Work Outside of the Public Service] *related challenges*’ (18 reports). Healthcare workforce challenges were also echoed in the unannounced facility visits. A patient at one clinic mentioned that “[t]*he* [HCWs] *are not enough sometimes. In most cases you arrive at a clinic at 5 am and you will be asked to leave unattended at 4 pm because there is just one professional nurse and she is not coping.*” Some participants at a community indaba also highlighted that “[t]*here was an exodus of operational managers of clinics due to attractive work opportunities elsewhere or in other provinces.*” Attendees at this indaba also opined that demotivation of employees was due to high workload, shortage of staff, and poor quality of healthcare services provided to the community.

### Information

Only one subtheme emerged under this a priori theme, namely ‘*poor information management*’ (26 reports). During a meeting with district employees, one of them mentioned that “[i]*nformation management is a problem in the districts because data capturers in clinics are also expected to do administrative work such as having to assist with the opening of new patient files.*”

### Medical products, vaccines and technologies

Four emerging subthemes were aligned with this a priori theme including: firstly, ‘*non-functional ICT* [information and communication technology] *system*’ (27 reports); secondly, ‘*shortage of resources/equipment*’ (26 reports); thirdly, ‘*pharmaceutical system challenges*’ (23 reports); and, finally, ‘*supply chain problems*’ (8 reports). A patient at one of the clinics stated that: “[t]*he current pharmacy assistant is sickly, but she still comes to work regardless. She has been ill from last year May or June and was at some point admitted to hospital. Her condition affects her mental well-being and it affects her work. The community does not feel safe when receiving medication dispensed by her because of fear that the wrong medication might be given to them. Patients feel safer when their medication is checked by the professional nurse.*”

### Financing

There were two emerging subthemes under this a priori theme: firstly, ‘*financial/cash-flow challenges*’ (39 reports) and, secondly, ‘*OSD* [Occupation Specific Dispensation] *implementation challenges*’ (2 reports). During a group discussion with managers, a senior official mentioned that “[t]*he lack of funding provided to district services and the insufficient budget provided to the department from the equitable share poses a huge risk as we cannot render services as well as we would like to*.”

### Leadership and governance

Six emerging subthemes were identified under this a priori theme: firstly, ‘*risk to patient care*’ (38 reports); secondly, ‘*poor leadership*’ (29 reports); thirdly, ‘*poor priority setting*’ (27 reports); fourthly, ‘*lack of governance structures*’ (22 reports); fifthly, ‘*lack of policy implementation*’ (8 reports); and, finally, ‘*non-appointment of hospital board members*’ (1 report). During a group meeting with health managers one indicated that “[m]*anagers in this department compromise the internal audit process by not responding timeously to audit report queries*”, while another stated that “[t]*he lack of integration of available funding is one of the most serious weaknesses within the* [DHS].”

### Causal loop diagram

**Fig. 2:**
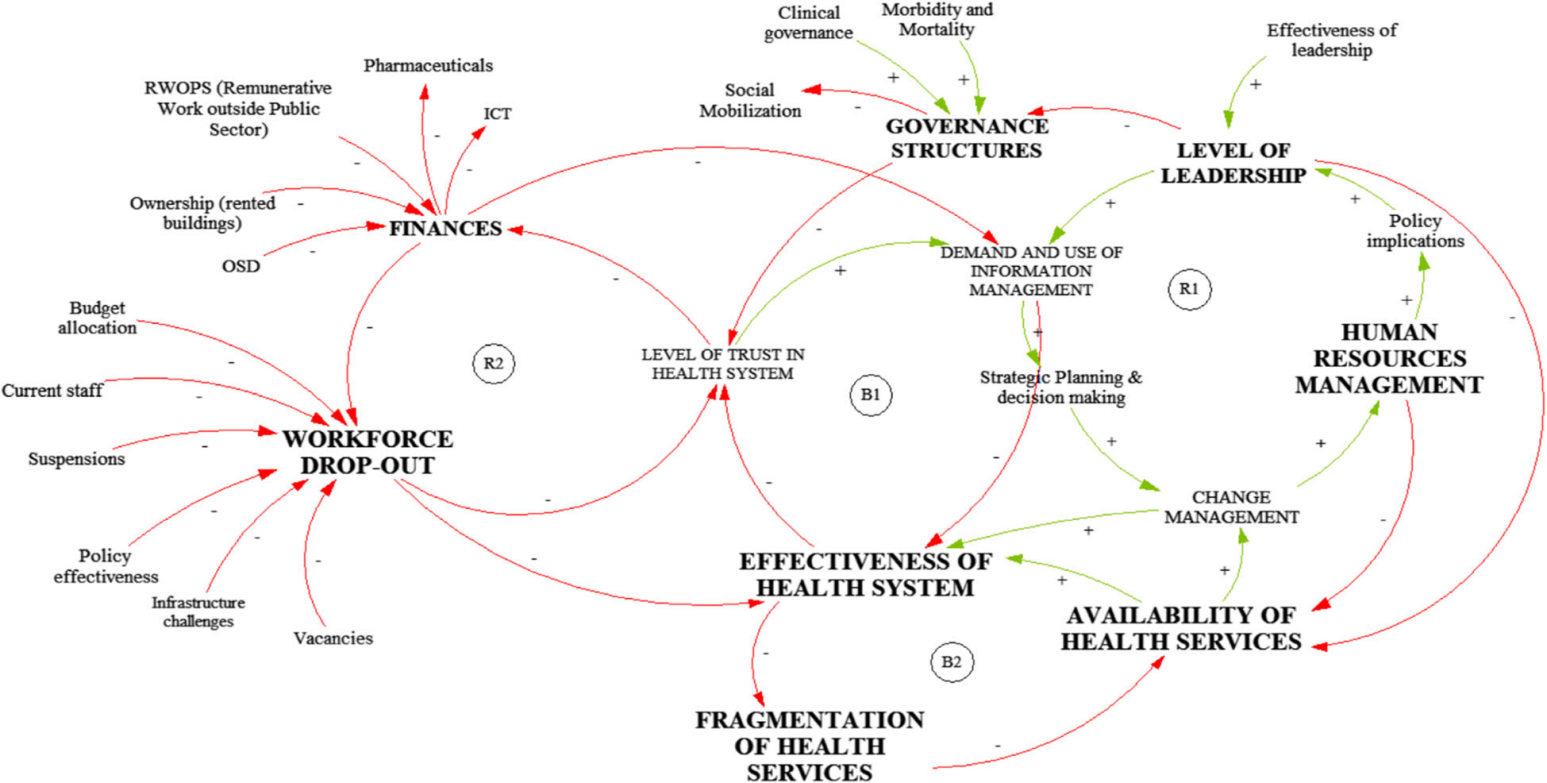
Causal loop diagram of public health system challenges affecting service delivery [35–37]

Figure 2 illustrates how components of the six building blocks framework affect (interact with) health system effectiveness. There are two reinforcing loops (R1 and R2) and two balancing loops (B1 and B2). The reinforcing loop, R1 indicates that with improved leadership, the demand for and use of health information increases. In turn, this leads to a need for continuous planning and decision making for improved performance in order to meet the demand for services. This again necessitates implementation of change management practices to enable adequate HRH infrastructure to implement policy amendments.

Balancing loop, B1 indicates that increases in the demand for services require that the health system should be effective in meeting those demands. When the demands are met, the level of community trust in the health system increases and thereby yields continuous patronage and use of the available healthcare services. This, in turn, leads to increased patient volumes that place higher demands on the system to perform at a level that meets expectations. When the health system is not performing in accordance with the demand, the use of the services again declines as a result.

Balancing loop, B2 indicates that decline in the effectiveness of the health system is compounded by fragmentation in the provision of healthcare services. This directly affects the vertical and horizontal integration of services and care at the facility level, thereby impacting on the quality and efficiency of service delivery. This again necessitates implementation of change management practices towards greater integration and improvement of services.

## Discussion

This situation appraisal sought to explore the health system dynamics associated with an (in) effective public health system in the Free State. A CLD was developed to illustrate the complex relationships among the various variables generated from the reports reviewed. The diagram demonstrated that a weakness in one variable has an effect on the others and therefore adequate healthcare provisioning depends on improved integration of and cooperation between the subsystems. It was therefore important to consider and apply system thinking as an approach when dealing with challenges in public health service delivery in the Free State.

The ensuing section discusses the challenges highlighted in the CLD in line with the WHO’s health system functional component or building block framework.

### Service delivery

Findings show that the stakeholders thought that the inefficiency of the health system was mainly influenced by fragmentation of health service provisioning. Fragmentation of healthcare services in the midst of the rising disease burden was cited as a major challenge in 42 of the 44 reports reviewed. Fragmentation of health services creates multifarious and often impersonal health systems that are challenging to navigate [39]. Particularly in African countries, the emergence of global health initiatives (GHIs) may have contributed to fragmentation by commonly operating in vertical ways and even bypassing existing country health systems [40]. A 2008 study exploring the extent of health systems fragmentation in three African countries (Ghana, South Africa and Tanzania), showed that South Africa had made the least progress in addressing fragmentation [41]. In 2010, it was recommended that the country should continue to request GHI funding for its ART programme, but that such funding should be used to address need across districts, services and time and should be effectively aligned with national policy and priorities – that is, ‘*a comprehensive and integrated approach*’ [42], p. 110.

Positive effects of the introduction of GHIs since 2000 have included greater stakeholder involvement and rapid expansion in HIV service delivery. However, the proportion of funding drawn from GHIs, even for the HIV and TB programmes, has been low in South Africa. The role of donor-funded, NGO-delivered services was critical in the initial stages of the ART roll-out plan, but has been curtailed over time. Negative effects of the GHIs may have included distorting recipient countries’ national policies, notably through detracting governments’ attention from efforts to strengthen health systems and “*‘re-verticalis* [ing]*’ planning, management and monitoring and evaluation systems*” [43], p. 239, [41]. Thereupon, the deployment of GHI resources in health systems in an integrated manner has been reported to yield positive results in Malawi, Ethiopia and Rwanda – particularly in relation to skills development and training of HCWs [44]. However, it is essential to augment capability at the national level to manage and deliver services, position interventions firmly within national strategies, ensure effective (integrated) implementation, and coordinate external support with local resources [45].

In the current study, the public health system was providing poor quality of care and yielding poor health outcomes due mainly to weak leadership and fragmentation of services at the implementation level. Leadership deficits and fragmentation of public healthcare services has also been observed in other South African studies [46–48] and research in Gauteng [49] showed that approximately 75% of randomly-selected participants indicated that they were no longer utilising public healthcare services due to the perceived diminished quality of these services. Thereupon, the 2014 Statistics South Africa general household survey indicated though that most (69%) households have a preference for public clinics and hospitals facilities first followed by a private doctor (29%) whenever a member falls ill [50], p. 28. The survey further showed that the Free State, ranked the third highest in terms of people ‘*very dissatisfied*’ with public facilities after Western Cape and North West, and ranked the highest in terms of people ‘*somewhat dissatisfied*’ with public facilities. In the current situation appraisal, stakeholders were prone to think that fragmentation and lack of coordination of public healthcare services and referral systems were major factors impacting on the quality of care.

Another challenge negatively affecting delivery of services in the current study was the lack of adequate security or alarm systems in the facilities for the safety of staff, patients and property. As highlighted during discussions with community members, there had been a large number of thefts and burglaries at the facilities, which called for the provincial health department to put measures in place to prevent reoccurrences and thereby place staff and patient safety first. Most of the clinics in the Province were fairly to very old and had unsuitable infrastructure to accommodate large volumes of patients. The infrastructure challenges and the lack of an organised and efficient booking system and timed appointments resulted in patients going to clinics very early in the morning and often being sent back home without receiving treatment. Longer waiting times were particularly experienced during rainy seasons and in the winter.

### Health workforce

A Brazilian study observed that disparity in the distribution of qualified health professionals was a major challenge resulting in unequal healthcare provision [51]. Similarly, an Ethiopian study observed that a motivated and committed health workforce was needed to expedite healthcare reform processes [52]. The problem of staff shortages was echoed in a Zambian study where patients were being attended by non-clinicians [53]. A Georgian study observed that where there was no clear vision and plans for HRH development, employees would inevitably leave to seek better opportunities elsewhere [54]. Another Ethiopian study showed job dissatisfaction and demotivation of the public healthcare workforce in public hospitals had potential impacts on the overall health system. Increased skill levels resulting from the provision of quality training, increases the level of service, which in turn increases the effectiveness of the health system [55].

As emphasised by stakeholders in the current situation appraisal, while the burden of disease had been increasing, the numbers of health professionals were decreasing in the Free State. It is of concern that even under these circumstances, the Free State Province failed to develop, nurture and integrate a community health worker cadre sufficient to provide support and alleviate the staff shortage pressures. This indicated that the health system strengthening in respect of HRH was needed in order to provide healthcare services to the population. Assigning a competent workforce that matches the patient load could decrease the likelihood of death or serious complications of patients and minimise diagnosis, care and treatment errors.

The challenge of unavailability of appropriate skills in the Free State was mainly due to resignation and suspensions as the provincial health department was experiencing leadership and HRH management challenges. Some participants also highlighted that there was an exodus of operational managers of clinics due to more attractive work opportunities elsewhere – in the private sector or in other provinces. It was thought that the Free State public health sector was unable to recruit as quickly as was required, failed to retain requisite skill, and that the local training institutions were also under-resourced implying a lack of opportunities for new HRH development. The participating stakeholders often associated HCW motivation with the provision of allowances, the level of safety of institutions and manageability of workloads.

### Information

A wide range of health information technology including equipment and know-how is in existence to generate, store and disseminate health information within healthcare systems, including electronic patient records, clinical databases, and financial and administrative systems [56]. However, a review and meta-synthesis of 20 studies in 11 countries identified operational challenges of DHIS2 including, inter alia, funding and HRH shortages, unmet training needs, inappropriate data, insufficient data security, and poor stakeholder communications [57].

In the current situation appraisal, stakeholders reported that requisite computer hardware and accompanying DHIS software were often not sufficiently provided at the local level. Patient record keeping was then done manually and this contributed to long patient waiting times and file losses and duplications. Searches for files for follow-up visits were cumbersome and resulted in new files being opened when old ones were not found. The capturing of prescriptions of medicines for patients at clinic pharmacies and medicine stock was also done manually due to lack of computer hardware and connectivity. Therefore, medicine tracking and stocktaking was done poorly and clinics frequently ran out of stock. This was exacerbated by a lack of adequate numbers of pharmacists and pharmacist’s assistants who could not be recruited because of lack of funds. Furthermore, the information that needed to be captured on DHIS had to be manually delivered first to the district offices for processing prior to submission to the corporate office for uploading on the DHIS. The capturing of prescriptions for medicines for patients at clinic pharmacies was also done manually. This resulted in an inability of pharmacists to track and trace previous quantities of medicine dispensed and patient adherence patterns.

Generally, disease tracking and reporting was thus unreliable. The local community health centres and some district hospitals were particularly negatively affected by unavailability of health information system hardware and software. In the main, these facilities also did not have reliable internet connectivity which constantly hindered transmission of information to the provincial and national health departments for monitoring of disease patterns and programme treatment outcomes. There was also increasingly a need for health information that would enable reduction of medical errors, improve cost efficiencies and improve the overall quality of public healthcare services.

### Medical products, vaccines and technologies

One derivative from the right to health is that all people have the right to medical products, vaccines and medical technologies including access to devices and procedures [58]. The UN Development Group (2003) defined access to affordable essential drugs on a sustainable basis as having continuous access to a minimum of 20 of the most essential drugs within 1 hour walk from the population [59], p. 89. The WHO defines a well-functioning health system as one that guarantees access to “*essential medical products, vaccines and technologies of assured quality, safety, efficacy and cost-effectiveness, and their scientifically sound and cost-effective use*” [9], p. 20. Nevertheless, there are many challenges resulting from the technology itself, end-users and environments that continue to undermine efforts to provide UHC in this regard. Research in three BRICS and Global South countries, China, India and South Africa, showed similar barriers to strengthening and developing health technology assessment systems such as lack of expertise, weak health data infrastructure, rising healthcare costs, fragmentation of healthcare systems and growth in non-communicable diseases [60].

In the current situation appraisal, stakeholders were of the opinion that medical product, vaccine and technology-related challenges particularly entailed lack of access and unaffordability, procurement, storage and distribution issues. Patients visiting public health facilities considered unavailability of personnel, medicines and devices to perform diagnostics as indicative of lack of access to services. Procurement of medicines was centralised at the provincial head office through a trading account operated by the medical depot on behalf of all the provincial hospitals and clinics. Medical products, medicines and medical device needs assessments were done at the peripheral facilities by the facility-based procurement officers who would capture the requirements on a Demand and Acquisition Plan for further processing by the provincial health department. The major challenge relating to some of the lifesaving medicines was that they were almost all under a transversal contract that was under the purview of the national health department. Provinces were not allowed to procure outside of these contracts. For the provinces to deviate from this system, they first would have had to request permission from the national health department. Sometimes permission would be granted particularly when a medicine shortage crisis was looming due to failure of the nationally-appointed suppliers to supply and deliver medicines and drugs that were ordered. Even though centralised procurement is considered to be a national pharmaceutical strength, the inefficiencies of suppliers’ performances created inefficient and unsatisfactory delivery of health services, medicines and other medical products. The situation was further compounded by shortages of appropriate pharmaceutical staff and ICT systems.

### Financing

Challenges identified by stakeholders in the current study under this a priori theme included insufficient health system financing, increasing costs, financial unsustainability and lack of financial autonomy. The provincial treasury allocates equitable share for health services based on the provincial government’s priorities. Health is currently the second priority following education [61].

The application of monetary inducements requires careful planning and management in order to avoid low staff morale [62]. The OSD was implemented in South Africa with the intention to increase the retention of HCWs in remote and rural areas through improved remuneration. However, stakeholders in the current situation appraisal suggested numerous problems with the implementation of the OSD policy, ranging from inadequate planning to budget overruns and some unintended negative consequences, including both doctors and nurses’ unmet expectations, inequalities in the amounts received, perceived unfairness and dissatisfaction, and divisions among the different categories of public service providers. When the OSD policy was implemented, this brought about an increase in costs of employee compensation inclusive of benefits and perks. Implementation of the OSD policy and instructions from the national public services and administration department created difficulties in respect of salary and grade progressions as the instructions were that corrections of salary and grade progressions had to be done retrospectively. This created budget pressures during the ensuing financial year to the extent that some critical health programmes had to be abandoned and others deferred.

The factors thought to cause inadequate financial resource allocation to meet the requirements for operating the health system included the high costs of OSD, RWOPS, office rental, pharmaceutical products and other consumables. Permission to do RWOPS for both the medical professionals and allied professions in particular, created huge staffing gaps against fixed salary payments for services not rendered, when it became exploited due to poor monitoring by management. RWOPS is a system created by the Public Service Act of 1994, Chapter VII, Section 30 [63]. This section empowers the political head of a department to give permission for qualifying employees to do remunerative work outside of their normal employment. Public service doctors, nurses and other professionals can thus be permitted to perform work in the private sector after their normal working hours. However, this situation got exploited due to poor and cumbersome monitoring. This, in turn, caused staff shortages, truancy, and misuse of public service equipment due to pursuit of additional remuneration by professionals.

Consequently, there was workforce dropout, resignation and early retirement due to limited budget allocation for HRH, unfilled vacancies, employee suspensions and high workloads – all of which negatively affected the availability of healthcare services. This stifled the effectiveness of the public health system and may have led to reduced levels of community trust in the system and decline in its use by the public health sector dependant population. As a result financial allocation to the public health sector was further reduced (R2). This reduction had to be managed by the provincial department itself through transferring funds from one financial account to another based on the month-by-month user statistics and re-direction of funds to high-pressure areas.

Health systems should raise adequate funds to ensure that citizens can use the services when needed and are protected from catastrophic expenditures and impoverishment that may follow. Proper budgeting processes should be embarked upon as informed by the data obtained from the DHIS, which should reflect and describe the disease patterns for better planning and decision making. In the Free State public health system this process was frustrated by unreliable data inputs from peripheral facilities due to lack of appropriate technology for record keeping and patient information. A compounding problem was the lack of financial management knowledge of the HCWs and managers which led to budgeting and resource-allocation challenges. As a result, the budgets were unreliable and under-budgeting of services commonly occurred. This led to instances where funds for budgeted commodities would run out in the middle of the financial year. The poor track record of under-budgeting and unreliable payment of suppliers led to some of the critical suppliers refusing to deliver orders of medicines and devices due to delayed payments and non-payments beyond the agreed 90-day period. This state of affairs was exacerbated by unnecessary hospital admissions and over-prescriptions of medicines by medical doctors, particularly those who were inexperienced.

### Leadership and governance

In the current situation appraisal, stakeholders thought that poor leadership and governance of the Free State public health sector was negatively impacting service delivery. This was thought to equate to ‘*risk to patient care*’ which was cited in 38 of the 44 reports. This perception was exacerbated by instances where patients felt that the staff attitude was unacceptable and reported that at times even patients requiring emergency attention were ignored.

Leadership and governance (or stewardship) of health systems is a complex yet critical building block of any health system [9]. The leadership and governance functions are crucial for equitable healthcare service provision across the public health system. Leadership and governance is however one of the most difficult functions of health systems to define, measure and monitor. This requires attention because without appropriate investment in the leadership and governance of health systems, any gains that are realised from investment in public health service delivery are unlikely to be sustained over the long term.

Citing Management Sciences for Health, Gilson and Daire emphasized that South African health managers must always be ‘*managers who lead*’ [64], p. 70. These authors argued that there were two main reasons why health system leadership is crucial: firstly, challenges of policy implementation and, secondly, challenges of organisational structures and culture. In the Free State, centralised decision-making without devolution of delegations to the DHS failed to provide the district and facility managers with the means to lead and manage. The situation appraisal observed that stakeholders believed that where there was weak leadership and poor governance in the presence of good policies but poor implementation, unsatisfactory priority setting and lack of governance structures to ensure accountability, the risk to patient care increased and provision of quality health service was compromised. The risk to patient care was further exacerbated by the absence of hospital boards in the majority of hospitals, and the fact that the clinic committees were weak to non-existent as they were mainly run by volunteers who attended meetings on an ad hoc basis. Also, the provincial health council had collapsed and the provincial AIDS council had been removed from the Premier’s office. Under weak leadership and poor governance, the culture of fragmented service provision – described as ‘*working alone together*’ [65] – continued.

The current study has an important limitation. The first author was the executive authority and the third author a senior manager employed by the Free State Department of Health during the time of the data gathering. Social desirability bias in the research subjects’ responses was however countered by exclusion of the political executive leader in most of the data gathering exercises, notably the investigations and interviews conducted by the DCSTs. The inclusion of co-authors who are not employees of the Free State Department of Health in the analysis and interpretation of the data further contributed to the objectivity of the multi-method appraisal. It is a strength of the study that it is one of the first to assess whole-system (as opposed to programme) related challenges using the WHO building blocks framework and the CLD methodology. Because a comprehensive definition of the public health system includes private and voluntary entities that contribute to the delivery of essential public health services within a jurisdiction [10], future research can explore the contribution of these sectors to overall public health systems performance.

## Conclusion

Despite political commitment, strong policies and sound regulations since 1994, the public health sector in South Africa and the Free State has not yielded desired health system performance due mainly to fragmentation and weak and lacklustre leadership at the district, regional and tertiary levels of care which are the implementation levels for health system reform to improve system performance. The stakeholders participating in this multi-method situation appraisal identified firstly, ‘*fragmentation of health services*’; secondly, ‘*staff shortages*’; and, thirdly, ‘*financial/cash-flow problems*’ as the major overall health system challenges resulting in non-availability and ineffectiveness of public health services in the Free State. There was a need for leadership reforms within and across the public healthcare service delivery platform in the Province – particularly to foster better integration of services and policy implementation.

The lack of integration of services due to fragmentation of system operations and poor policy coordination had exacerbated verticalisation and encouraged a ‘*solo*’ mentality and ‘*bypassing*’ of existing policies and procedures thereby causing poor health system performance. Stakeholders considered this a major contributing factor to poor disease and treatment outcomes. Several HRH issues, as well as poor leadership and governance, emerged as among the main contributory factors to poor public health service delivery.

The findings of this multi-method appraisal suggest that for efficient and effective public health system performance to be realised, good leadership and governance had to be entrenched as a matter of urgency. Allocation of sufficient human and material resources was necessary and a strategic intervention centered on improving and enhancing integration of public healthcare services had to be implemented in order to improve the Free State’s public health system’s performance and population health outcomes. Multi-method appraisal of public health system performance may also be useful to inform health systems strengthening in other provinces of South Africa and in other resource-constrained public health settings. Our study illustrates the need for research to focus on whole-system deficiencies and changes towards better-performing integrated public health systems.

## Data Availability

The dataset analysed during the current study is available from PC on reasonable request.

## Abbreviations

AIDS: Acquired Immunodeficiency Syndrome
ANC: African National Congress
CEO: Chief Executive Officer
CLD: Causal Loop Diagram
DCST: District Clinical Specialist Team
DHC: District Health Council
DHIS: District Health Information System
DHS: District Health System
EMS: Emergency Medical Service
GHI: Global Health Initiative
HCW: Healthcare worker
HIV: Human Immunodeficiency Virus
HRH: Human Resources for Health
ICT: Information and Communication Technology
NGO: Non-governmental Organisation
OSD: Occupation Specific Dispensation
PHC: Primary Health Care
RWOPS: Remunerative Work Outside of the Public Service
TB: Tuberculosis
UHC: Universal Health Care
WHO: World Health Organization
